# Gd_2_O_3_/b‐TiO_2_ composite nanoprobes with ultra‐high photoconversion efficiency for MR image‐guided NIR‐II photothermal therapy

**DOI:** 10.1002/EXP.20220014

**Published:** 2022-06-13

**Authors:** Jia Chen, Tianxiang Chen, Qianlan Fang, Chunshu Pan, Ozioma Udochukwu Akakuru, Wenzhi Ren, Jie Lin, Aizhu Sheng, Xuehua Ma, Aiguo Wu

**Affiliations:** ^1^ Cixi Institute of Biomedical Engineering, International Cooperation Base of Biomedical Materials Technology and Application, Chinese Academy of Science (CAS) Key Laboratory of Magnetic Materials and Devices and Zhejiang Engineering Research Center for Biomedical Materials Ningbo Institute of Materials Technology and Engineering, CAS Ningbo China; ^2^ School of Life Science and Engineering Southwest Jiaotong University Chengdu China; ^3^ Advanced Energy Science and Technology Guangdong Laboratory Huizhou China; ^4^ University of Chinese Academy of Sciences Huairou Beijing China; ^5^ Department of Radiology, Hwa Mei Hospital University of Chinese Academy of Sciences Ningbo China

**Keywords:** black‐titanium dioxide, magnetic resonance imaging, photothermal therapy, polydopamine, second near‐infrared

## Abstract

Photothermal therapy (PTT), as an important noninvasive and effective tumor treatment method, has been extensively developed into a powerful cancer therapeutic technique. Nevertheless, the low photothermal conversion efficiency and the limited tissue penetration of typical photothermal therapeutic agents in the first near‐infrared (NIR‐I) region (700–950 nm) are still the major barriers for further clinical application. Here, we proposed an organic/inorganic dual‐PTT agent of synergistic property driven by polydopamine‐modified black‐titanium dioxide (b‐TiO_2_@PDA) with excellent photoconversion efficiency in the second NIR (NIR‐II) region (1000–1500 nm). More specifically, the b‐TiO_2_ treated with sodium borohydride produced excessive oxygen vacancies resulting in oxygen vacancy band that narrowed the b‐TiO_2_ band gap, and the small band gap led to NIR‐II region wavelength (1064 nm) absorbance. Furthermore, the combination of defect energy level trapping carrier recombination heat generation and conjugate heat generation mechanism, significantly improved the photothermal performance of the PTT agent based on b‐TiO_2_. The photothermal properties characterization indicated that the proposed dual‐PTT agent possesses excellent photothermal performance and ultra‐high photoconversion efficiency of 64.9% under 1064 nm laser irradiation, which can completely kill esophageal squamous cells. Meanwhile, Gd_2_O_3_ nanoparticles, an excellent magnetic resonance imaging (MRI) agent, were introduced into the nanosystem with similar dotted core–shell structure to enable the nanosystem achieve real‐time MRI‐monitored cancer therapeutic performance. We believe that this integrated nanotherapeutic system can not only solve the application of PTT in the NIR‐II region, but also provide certain theoretical guidance for the clinical diagnosis and treatment of esophageal cancer.

## Graphical abstract

Low photoconversion efficiency and limited tissue penetration are the major barriers for photothermal therapy (PTT) agent application. Here, an efficient organic/inorganic dual‐PTT agent guided by MRI was proposed. It is proved the oxygen vacancy band narrowed its band gap that led to NIR‐II region absorb. Furthermore, the defect energy level trapping carrier and conjugate synergistic heat generation result in high photoconversion efficiency.

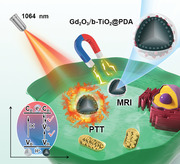

## 1 INTRODUCTION

Photothermal therapy (PTT), as a novel cancer treatment method with high selectivity, minimal invasiveness, and strong operability, has been extensively developed into a powerful cancer therapeutic technique over the past decades. In particular, photothermal therapeutic agents can locally generate hyperthermia to ablate tumor cells in the diseased area when activated by a special wavelength.^[^ ^1^ ^]^ Titanium dioxide (TiO_2_) has been regarded as a favorable photothermal agent for PTT, attributed to its excellent photocatalytic property, high photostability, and favorable biocompatibility. However, TiO_2_ can only absorb energy in the ultraviolet region (272–390 nm) owing to its large band gap (∼3.0 eV).^[^ ^2^ ^]^ Ultraviolet light lacks deep tissue penetration and poses side effects to normal tissues, thus greatly limiting the application of TiO_2_ in the clinic. Therefore, many efforts have been made to further expand its absorption range from the ultraviolet to the near‐infrared (NIR) region, such as hydrogenation reduction,^[^ ^3^ ^]^ doping,^[^ ^4^ ^]^ and other methods ^[^ ^5^ ^]^ to optimize its electronic energy level structure, shorten the forbidden band width, and improve optical performance. For instance, Ren et al. first presented a black hydrogenated TiO_2_ (H‐TiO_2_), where white TiO_2_ reacted with hydrogen plasma at a high‐power density introducing Ti^3+^ and oxygen defect that turned it into black H‐TiO_2_.^[^ ^6^ ^]^ Consequently, the black H‐TiO_2_ exhibited NIR light‐triggered photothermal performance with excellent photothermal conversion for tumor therapy, which was referred to its dramatically enhanced nonradiative recombination. In order to achieve diagnostic and therapeutic multi‐functions, Wang et al. synthesized a Fe@γ‐Fe_2_O_3_@H‐TiO_2_ nanocomposite by one‐step hydrogen reduction that showed outstanding photoconversion efficiency under 808 nm laser irradiation resulting from the narrow band gap (1.971 eV) of H‐TiO_2_ and multiple circuit loops for electron transitions between H‐TiO_2_ and γ‐Fe_2_O_3_.^[^ ^7^ ^]^ Notably, the nanocomposite possessed magnetic resonance imaging, PTT, and tumor targeting functions. It is noteworthy that the photothermal therapeutic agents based on black TiO_2_ (b‐TiO_2_) nanocomposites are mainly applied in the first NIR (NIR‐I) region (700–950 nm), which requires a high laser density (2 W cm^−2^) due to the limitation of light absorption and scattering by tissues.^[^ ^8^ ^]^ However, this laser density exceeds the maximum allowable skin exposure set by the American National Standards Institute (i.e., 0.33 W cm^−2^ under 808 nm laser irradiation),^[^ ^9^ ^]^ and has potential damages to the surrounding normal tissues. Hence, the PTT therapeutic efficacy and safety of b‐TiO_2_ need to be further improved.

Recently, the development of b‐TiO_2_‐based photothermal therapeutic agents responsive in the second NIR (NIR‐II) region (1000–1500 nm) has garnered significant interest, due to long wavelength light with higher maximum allowable skin exposure (i.e., 1 W cm^−2^ under 1064 nm laser irradiation) and deeper tissue penetration depth because of less tissue scattering.^[^ ^10^ ^]^ For example, Guo et al. used mild hydrogenation with NaBH_4_ method to synthesize b‐TiO_2_ with abundant oxygen vacancies on its surface that display high photoconversion efficiency of 29.44% in the NIR‐II region, which exhibit precise potential for photoacoustic image‐guided tumor‐targeted PTT.^[^ ^11^ ^]^ Han et al. constructed core/shell‐structured b‐TiO_2‐_ *_x_* nanocomposites via aluminum‐reduction method.^[^ ^12^ ^]^ The results showed that oxygen‐deficient TiO_2–_ *_x_* layer can also enhance the photothermal conversion efficiency (39.8%) of b‐TiO_2_ nanoparticles under 1064 nm laser for photothermal hyperthermia. These studies successfully developed the photothermal agents based on b‐TiO_2_ to be excitable in the NIR‐II region and effectively caused the death of cancer cells under photothermal treatment, which solved the drawback of shallow tissue penetration and low maximum permissible exposure. However, the existing b‐TiO_2_‐based photothermal therapeutic agents generally show a very broad absorption and low photothermal conversion efficiencies. Consequently, it is urgent to develop b‐TiO_2_‐based photothermal therapeutic agents with excellent photoconversion efficiency in the NIR‐II region, which can alter more light energy to thermal energy for efficient PTT.^[^ ^13^ ^]^

Dopamine, a neurotransmitter in the human brain, has good biocompatibility and easily oxidation and self‐aggregated to form polydopamine (PDA) under alkaline conditions (pH = 8.5).^[^ ^14^ ^]^ PDA nanoparticles exhibit excellent photothermal conversion properties and are emerging as a novel PTT agent for tumor. Specifically, the polymer contains a π‐π conjugated structure, similar to many aromatics and conjugated molecules, which is conducive to the migration of free electrons and carriers, so that it has broad‐spectrum absorption of light in the ultraviolet‐near infrared region.^[^ ^15^ ^]^ Based on this, we believe that combining PDA with b‐TiO_2_ can not only improve the biocompatibility of the photothermal therapeutic agent based on b‐TiO_2_ nanoparticles, but also significantly improve its photo‐absorption performance and photothermal conversion capacity in the NIR‐II region through the synergistic photothermal effect of b‐TiO_2_ and PDA, to achieve a highly efficient photothermal therapeutic agent for tumors. Meanwhile, the single diagnosis or treatment strategies have been difficult to achieve the clinical requirements. The combination of various diagnosis and therapy technologies to build a diagnosis and treatment platform is the key research direction currently.^[^ ^16^ ^]^ Therefore, gadolinium oxide (Gd_2_O_3_) with appreciable biocompatibility and magnetic resonance (MR) imaging performance can be introduced into b‐TiO_2_ that realizes the MR image‐guided therapy for cancer.^[^ ^17^ ^]^

In this work, b‐TiO_2_ was prepared by one‐step hydrogenation reduction method, while Gd_2_O_3_/b‐TiO_2_ composite nanoparticles with dotted core‐shell were prepared by polyol method. On this basis, Gd_2_O_3_/b‐TiO_2_@PDA nanoprobes with core‐shell structure were obtained by the oxidative self‐aggregation of dopamine hydrochloride. The nanoprobes not only extended the light absorption of b‐TiO_2_ to 1064 nm that solved the shallow tissue penetration depth in NIR‐I region, but also provided extremely high photoconversion efficiency of 64.9% at the laser irradiation density of 1 W cm^−2^ that evaded the irradiation laser density exceeding maximum allowable skin exposure. In addition, the constructed nanoprobes were applied to esophageal squamous cell carcinoma cancer model, and the results showed that the nanoprobes could offer high‐signal MR imaging on the tumor region and had a significant therapeutic effect on the tumor cells. Further, the application of these multifunctional nanoprobes not only reflects the advantages of integrated diagnosis and treatment technology at the NIR‐II region, but also promotes the research on the diagnosis and treatment technology of esophageal squamous cell carcinoma.

## 2 RESULTS AND DISCUSSION

### 2.1 Synthesis and characterization of Gd_2_O_3_/b‐TiO_2_@PDA

**SCHEME 1:**
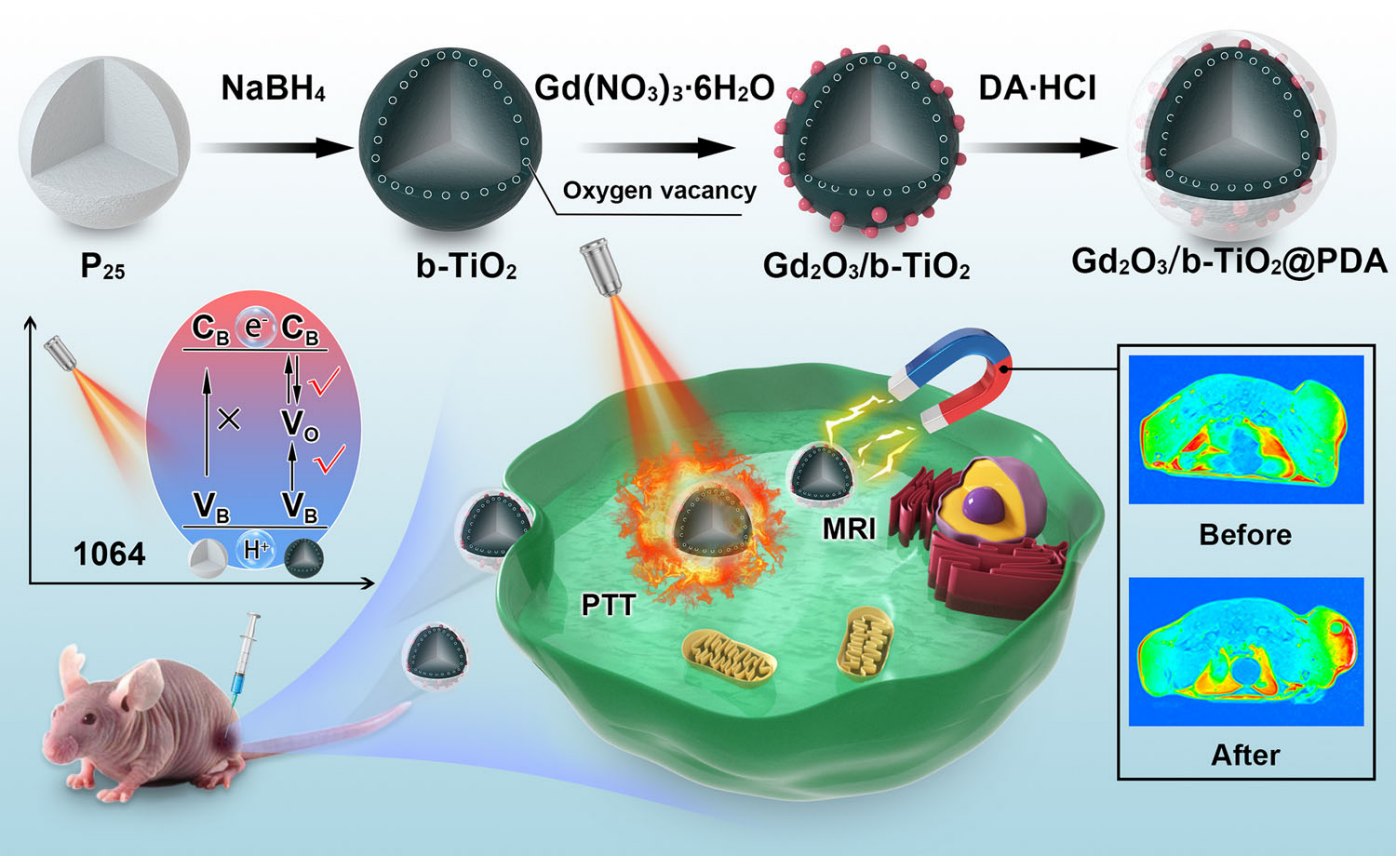
Schematic illustration of the synthetic route to Gd_2_O_3_/b‐TiO_2_@PDA nanoprobes and their application for MR image‐guided NIR‐II photothermal therapy

**FIGURE 1:**
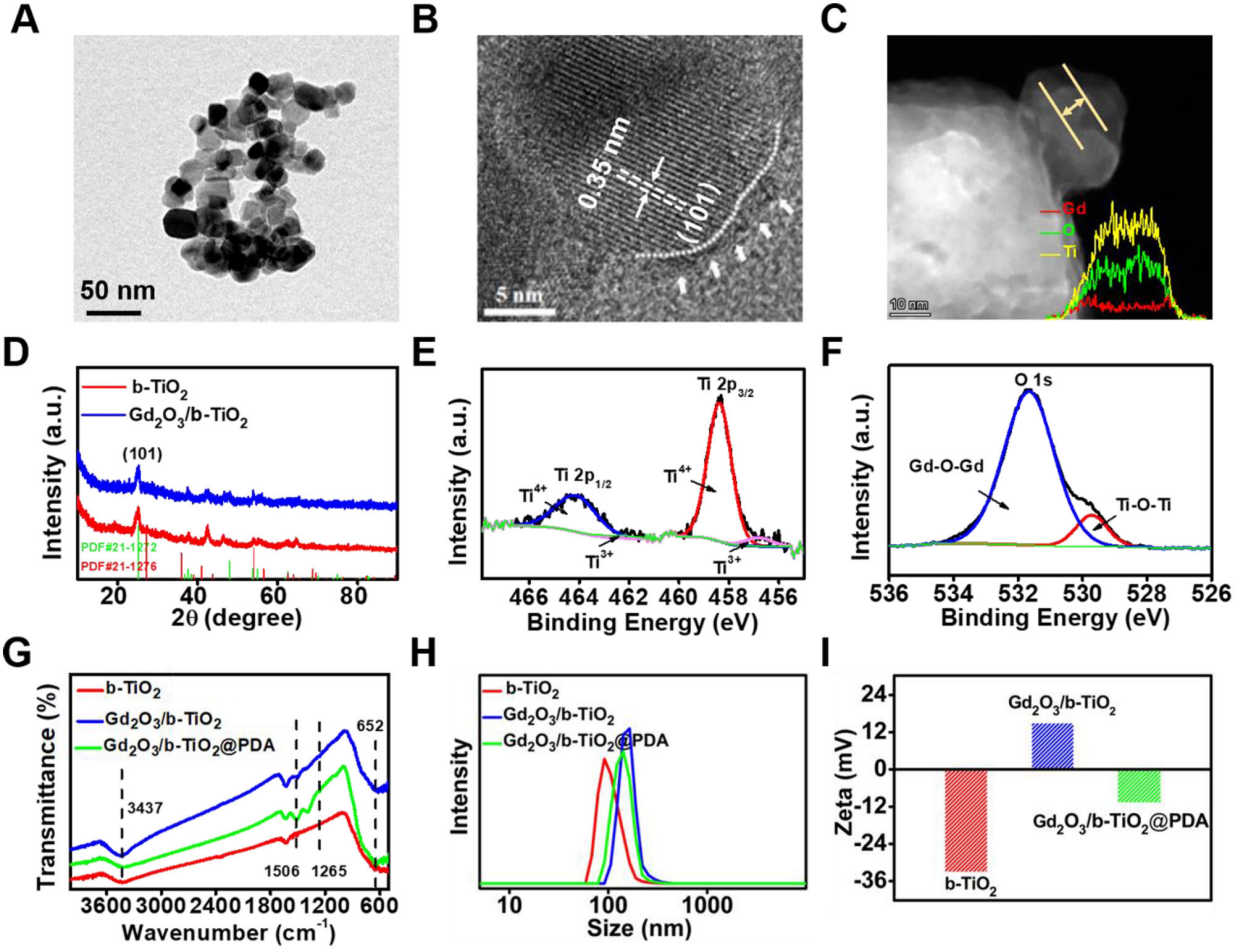
(A) TEM b‐TiO_2_. (B) HRTEM image of the b‐TiO_2_ with crystalline core‐amorphous shell structure. The crystalline core with well‐resolved lattice fringes and the interplanar distance consistent with the (101) planes of anatase phase TiO_2_. (C) TEM image of Gd_2_O_3_/b‐TiO_2_ (Inset: the corresponding EDS line scanning). (D) XRD patterns of b‐TiO_2_ and Gd_2_O_3_/b‐TiO_2_. (E) XPS spectra of Ti (2p) in Gd_2_O_3_/b‐TiO_2_. (F) XPS spectra of O (1S) in Gd_2_O_3_/b‐TiO_2_. (G) FT‐IR spectra of b‐TiO_2_, Gd_2_O_3_/b‐TiO_2_, and Gd_2_O_3_/b‐TiO_2_@PDA. (H) DLS of b‐TiO_2_, Gd_2_O_3_/b‐TiO_2_, and Gd_2_O_3_/b‐TiO_2_@PDA. (I) Zeta potentials of b‐TiO_2_, Gd_2_O_3_/b‐TiO_2_, and Gd_2_O_3_/b‐TiO_2_@PDA

The synthetic route of the Gd_2_O_3_/b‐TiO_2_@PDA nanoparticles is shown in Scheme 1. A solid‐phase reduction reaction was used to acquire the b‐TiO_2_ nanoparticles, on this basis, Gd_2_O_3_/b‐TiO_2_ nanoparticles were prepared by a polyol method. In order to increase the biocompatibility of the materials, Gd_2_O_3_/b‐TiO_2_@PDA nanoparticles were obtained by oxidative self‐aggregation of dopamine hydrochloride. As shown in HRTEM image (Figure 1B), the crystalline core‐amorphous shell structure can be clearly identified, that the crystalline core exhibited well‐resolved lattice fringes of 0.35 nm corresponding to the (101) planes of anatase phase TiO_2_ (Figure 1D).^[^ ^18^ ^]^ The TEM image showed that the morphology of Gd_2_O_3_/b‐TiO_2_ (Figure S1) had no significantly change compared with b‐TiO_2_ (Figure 1A). The DLS data (Figure 1H) showed that the average hydrodynamic particle size of Gd_2_O_3_/b‐TiO_2_ was increased from 93.3 to 155.7 nm when Gd_2_O_3_ was loaded, which was consistent with the TEM results. As shown in Figure 1C, the EDS line scanning and the HAADF‐STEM images indicated that the element of Ti and O were uniformly distributed and the element of Gd was concentrated on the surface of b‐TiO_2_, which revealed that the Gd_2_O_3_/b‐TiO_2_ was successfully fabricated. Moreover, the TEM images revealed that the as‐synthesized Gd_2_O_3_/b‐TiO_2_ was coated by the PDA shell with thin layer after self‐polymerization of DA·HCl (Figure S2). In addition, the DLS measured the polymer dispersity index (PDI) of b‐TiO_2_, Gd_2_O_3_/b‐TiO_2_ and Gd_2_O_3_/b‐TiO_2_@PDA are 0.254, 0.242, and 0.172, which suggested that the nanoparticles exhibited excellent dispersion in aqueous solutions. The smaller PDI can explain the average hydrodynamic size of Gd_2_O_3_/b‐TiO_2_@PDA smaller than Gd_2_O_3_/b‐TiO_2_, indicating the nanoparticles with better dispersion after PDA coating.

Furthermore, the crystalline structure of b‐TiO_2_ and Gd_2_O_3_/b‐TiO_2_ were characterized by XRD (Figure 1D). It can be observed that the anatase and rutile crystal structures were both maintained in b‐TiO_2_, the highest peak observed at 25.3° corresponded to the characteristic peak (101) of anatase phase TiO_2_ (JCPDS, PDF#21‐1272), the weak peaks belonged to the rutile phase TiO_2_ (JCPDS, PDF#21‐1276), indicating the anatase crystal structures was the majority phase in b‐TiO_2_ and Gd_2_O_3_/b‐TiO_2_. In addition, there were no obvious characteristic peaks in XRD spectrum, representing that the Gd_2_O_3_ existed in Gd_2_O_3_/b‐TiO_2_ nanoparticles in the amorphous form because of the small size.^[^ ^19^ ^]^ The Gd (3d) XPS spectra of the Gd_2_O_3_/b‐TiO_2_ showed that the characteristic peaks of Gd 3d_3/2_ and 3d_5/2_ at 1219.78 and 1187.42 eV, respectively, corresponding to the different binding energies with different spin orbits of Gd in Gd_2_O_3_ (Figure S4). Meanwhile, the XPS spectra of O (1s) in the Gd_2_O_3_/b‐TiO_2_ presented the Gd‐O peak at 531.2 eV, confirming the existence of Gd_2_O_3_ in the Gd_2_O_3_/b‐TiO_2_ nanoparticles (Figure 1F).^[^ ^20^ ^]^

Next, the functional groups on the surface of the nanoparticles were employed by FT‐IR. Concretely, the characteristic peaks at 652 and 3437 cm^−1^ were attributed to the Ti‐O‐Ti and O‐H of b‐TiO_2_, and the characteristic peaks below 900 cm^−1^ were assigned to the Gd‐O stretching in Gd_2_O_3_.^[^ ^21^ ^]^ In addition, the characteristic peaks at 1265 and 1506 cm^−1^ belonged to the groups of C‐H and N‐H (‐NH_2_) in PDA, respectively (Figure 1G).^[^ ^22^ ^]^ The corresponding zeta potential results are shown in Figure 1I. The zeta potential of b‐TiO_2_ was −33 mV, attributed to the ‐OH groups, while that for Gd_2_O_3_/b‐TiO_2_ was +15 mV because the Gd_2_O_3_ was positively charged. The zeta potential of Gd_2_O_3_/b‐TiO_2_@PDA was −10.6 mV, attributed to the ‐OH groups. The change of zeta potential also further proved that the Gd_2_O_3_/b‐TiO_2_@PDA nanoprobes were successfully prepared.

### 2.2 Characterization of photothermal mechanism

**FIGURE 2:**
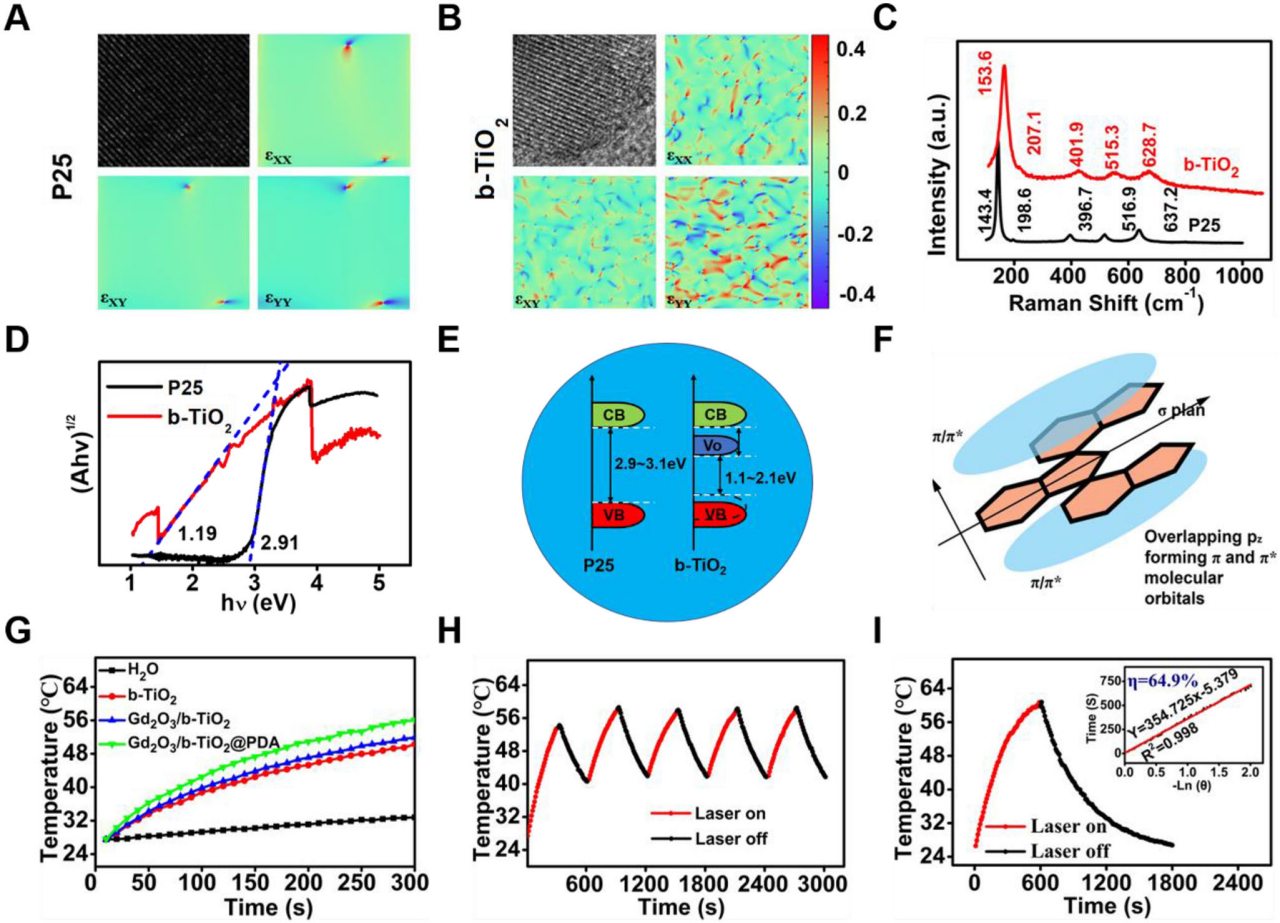
GPA of (A) P25 and (B) b‐TiO_2_. (C) Raman spectra of P25 and b‐TiO_2_. (D) Optical bandgaps of P25 and b‐TiO_2_. (E) Energy band structure of P25 and b‐TiO_2_. (F) Schematic illustration of the π molecular orbitals in conjugated polymer. (G) Temperature change curves of b‐TiO_2_, Gd_2_O_3_/b‐TiO_2_, and Gd_2_O_3_/b‐TiO_2_@PDA (b‐TiO_2_: 200 μg mL^−1^) under 1064 nm laser irradiation at intensity of 1 W cm^−2^ for 5 min. (H) Heating curve of Gd_2_O_3_/b‐TiO_2_@PDA dispersed in water for five cycles under 1064 nm laser irradiation. (I) Temperature ascending and descending curve of Gd_2_O_3_/b‐TiO_2_@PDA response to 1064 nm laser on and off in period of 2400 s. Inset: Linear time data versus −ln θ obtained from the descending stage

In order to characterize the defect, multiple test methods were used, such as Geometric‐phase analysis (GPA), Raman spectroscopy, and XPS. Specifically, the GPA result indicated that the P25 disorder degree was approximately zero and the image color was uniform, indicating that the crystal form of the particles was complete and the atomic arrangement was highly ordered (Figure 2A). However, the GPA result of b‐TiO_2_ indicated that the degree of disorder was relatively high and the color of the image results was discontinuous, indicating that the edge part of the b‐TiO_2_ nanoparticles (the core‐shell junction) with a poor crystal form and the atomic arrangement was in a highly disordered state (Figure 2B). The results of Raman spectroscopy illustrated that the characteristic peaks of b‐TiO_2_ had a small deviation and the peak width was significantly increased compared with P25 nanoparticles (Figure 2C), mainly because of the production of oxygen vacancies and emergence of low‐priced Ti^3+^.^[^ ^23^ ^]^ In addition, the presence of Ti^3+^ could be clearly substantiated by the XPS results of Ti_2P_ in b‐TiO_2_ and Gd_2_O_3_/b‐TiO_2_ nanoparticles (Figure S3 and Figure 1E), which confirmed the conclusion of Raman spectroscopy.^[^ ^24^ ^]^

By analyzing the results of the diffuse reflectance UV‐vis spectra of P25 and b‐TiO_2_, it can be obtained that the band gap widths of P25 and b‐TiO_2_ were 2.91 and 1.19 eV, respectively (Figure S5 and Figure 2D).^[^ ^25^ ^]^ More importantly, the absorption wavelength threshold value was 1240/1.19 nm, which showed that the nanoprobes can be excited by the NIR‐II region laser. In order to study the photothermal mechanism, the energy band structures of P25 and b‐TiO_2_ were analyzed. As shown in Figure 2E, ordinary P25 cannot be excited by NIR laser due to the large forbidden band width. However, the obtained b‐TiO_2_ nanoparticles produced the oxygen vacancy band below the conduction band and the valence band shifted upward, indicating the b‐TiO_2_ exhibited multiple bands that narrowed its band gap. When the b‐TiO_2_ was excited under the laser, the electrons located in the valence band accepted energy transition to the vacancy band, and then the first‐excited electrons can absorb smaller energy transferred to the conduction band, led to the light absorb wavelength of b‐TiO_2_ shifting to the NIR‐II window. After the second‐excited, the recombination of the electron–hole pairs occurred, which cannot be prevented. Most notably, the oxygen vacancies have a strong electron trapping ability, so they are generally used as the recombination center of electron–hole pairs in semiconductor catalysts.^[^ ^26^ ^]^ The energy generated by the recombination process will be de‐excited in the form of thermal radiation and converted into thermal energy. Meanwhile, the surface PDA is an analogue of conjugated polymer, and its possible π molecular orbital theoretical model is shown in Figure 2F. The ground state of the molecule corresponded to the highest occupied molecular orbital, which was equivalent to the valence band. The excited state of the molecule corresponded to the highest unoccupied molecular orbital, which was equivalent to the conduction band. More importantly, charge carriers can move between the PDA and b‐TiO_2_ that brought multiple electron transfer circuits and narrow band gaps, which may be beneficial for the nanocomposites to convert more light energy into thermal energy.^[^ ^15^ ^]^ On this basis, the photothermal mechanism of the Gd_2_O_3_/b‐TiO_2_@PDA composite probes had been fully explained, which corresponded to its excellent photothermal performance in the NIR‐II region.

The photothermal performances of b‐TiO_2_, Gd_2_O_3_/b‐TiO_2_, and Gd_2_O_3_/b‐TiO_2_@PDA were measured under 1064 nm laser irradiation. As shown in Figure 2G, the temperature of b‐TiO_2_, Gd_2_O_3_/b‐TiO_2_ and Gd_2_O_3_/b‐TiO_2_@PDA nanoparticles increased by 22.7°C, 24.4°C, and 28.5°C, respectively. Compared with b‐TiO_2_, the temperature of Gd_2_O_3_/b‐TiO_2_@PDA significantly increased. The results of the three materials were consistent with their UV–vis spectrum results and the extinction coefficient obtained from the absorption spectrum is 4.74 g^−1^ cm^−1^, which also means that the material had excellent photothermal performance (Figure S7). In addition, the temperature showed a regular gradient increase with various concentrations of Gd_2_O_3_/b‐TiO_2_@PDA and laser power density. The photothermal heating curves of different concentrations of b‐TiO_2_ and Gd_2_O_3_/b‐TiO_2_ were shown in Figure S6, and the trend was basically same, but the heating effect was lower than the Gd_2_O_3_/b‐TiO_2_@PDA nanoprobes. At the same time, the temperature change corresponding to the laser on/off repeatedly for 5 times indicated that the Gd_2_O_3_/b‐TiO_2_@PDA nanoprobes offered considerable photothermal stability (Figure 2H). On this basis, the heating and cooling curve of the material was measured, and the photothermal conversion efficiency was 64.9% (Figure 2I).

### 2.3 MR imaging performance characterization

**FIGURE 3:**
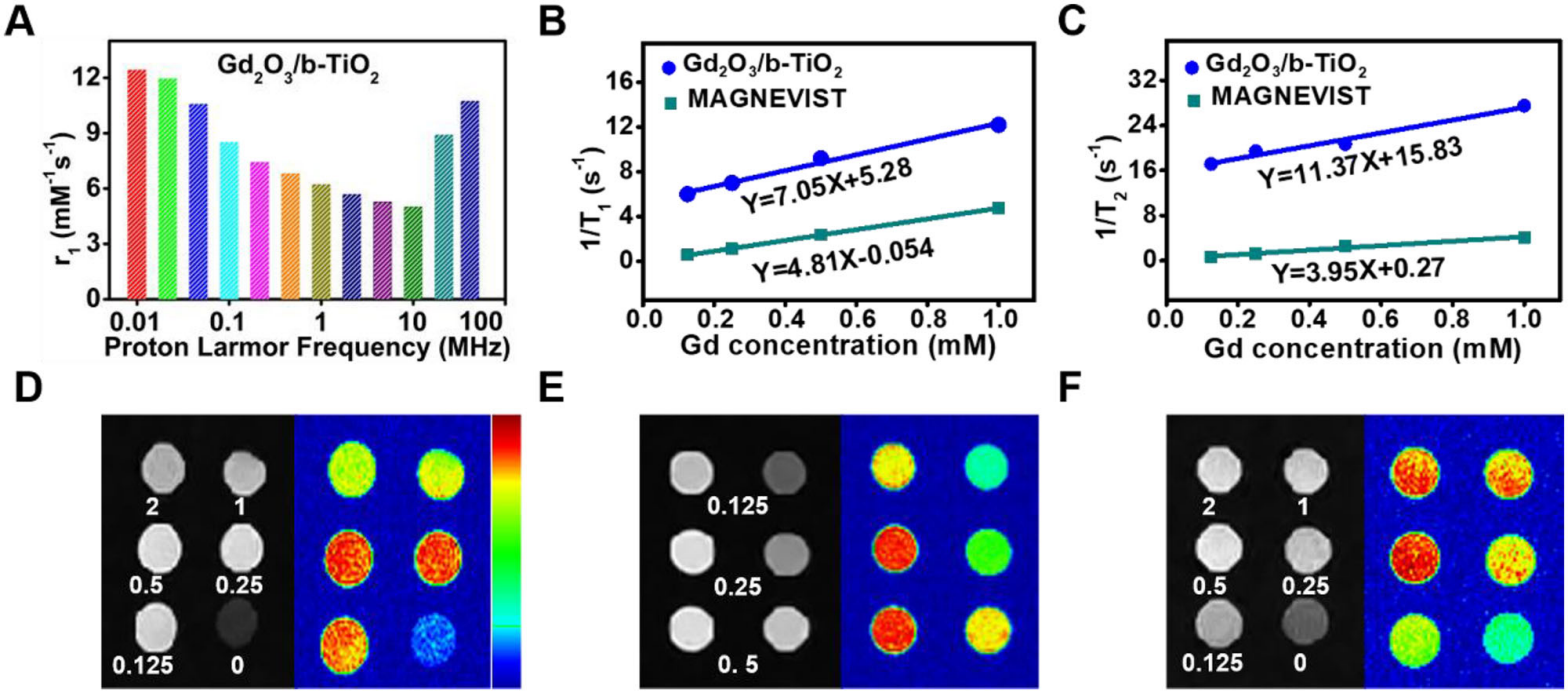
(A) Nuclear magnetic relaxation dispersion profile of Gd_2_O_3_/b‐TiO_2_ at 25°C. (B) T_1_ relaxation curve of Gd_2_O_3_/b‐TiO_2_ and MAGNEVIST. (C) T_2_ relaxation curve of Gd_2_O_3_/b‐TiO_2_ and MAGNEVIST. (D) T_1_‐weighted MR images of Gd_2_O_3_/b‐TiO_2_ with different concentrations of Gd^3+^ (mM). (E) T_1_‐weighted MR images of Gd_2_O_3_/b‐TiO_2_ and MAGNEVIST at low concentrations (left: Gd_2_O_3_/b‐TiO_2_, right: MAGNEVIST). (F) T_1_‐weighted MR images of Gd_2_O_3_/b‐TiO_2_@PDA with different concentrations of Gd^3+^ (mM)

Here, in order to characterize the imaging performance, the distribution of Gd elements on the surface of the material was determined by EDS line scan (Figure 1C). In the selected area, the content of Ti and O elements presented an isosceles trapezoid distribution, because b‐TiO_2_ had a solid structure. However, Gd_2_O_3_ presented an approximate horizontal line, indicating that the Gd element was only uniformly distributed on the surface of the nanoprobes. These results indicated that the characteristic structure of a dotted core‐shell may be formed, which may affect MR imaging performance by changing the number of bound water proton.^[^ ^27^ ^]^ The nuclear magnetic relaxation dispersion profile is shown in Figure 3A, which expressed the corresponding longitudinal relaxation rate in a certain frequency range.

The molar longitudinal relaxivity r_1_ and transverse relaxivity r_2_ were calculated from the slope of the linear relationships of relaxation time (1/T_1_, 1/T_2_) and concentration of Gd, which represented the efficiency of the nanoprobes as contrast agents. As shown in Figures 3B and 3C, the r_1_ values of Gd_2_O_3_/b‐TiO_2_ and MAGNEVIST were 7.05 and 4.81 mM^−1^ s^−1^, respectively. The r_1_ value of Gd_2_O_3_/b‐TiO_2_ was much higher than that of MAGNEVIST, indicating that Gd_2_O_3_/b‐TiO_2_ had a stronger shortening T_1_ effect and more suited for positive MRI contrast agents. The r_2_/r_1_ value of Gd_2_O_3_/b‐TiO_2_ was 1.61, indicating that the Gd_2_O_3_/b‐TiO_2_ nanoparticles had excellent T_1_ MR imaging performance. The T_1_‐weighted MR imaging showed that the imaging signal was strongest when the concentration of Gd at 0.5 mM (Figure 3D,E). Due to the T_1_‐weighted MR imaging performance being influenced by the sum of r_1_ and r_2_ relaxivity, when the concentration of Gd exceeds a certain limit, the MR imaging performance is largely related to r_2_ relaxivity, causing negative (dark) contrast compared with higher concentration of Gd in MR images. Compared with the commercial MAGNEVIST, the nanoprobes had a stronger imaging signal at low concentrations, which indicated the nanoprobes had higher safety. Meanwhile, the MR imaging signal of Gd_2_O_3_/b‐TiO_2_@PDA was highly consistent with that of the Gd_2_O_3_/b‐TiO_2_, indicating that the PDA encapsulation does not affect the imaging performance (Figure 3F). The excellent MR imaging performance of nanoprobes can realize the function of real‐time monitoring.

### 2.4 Cellular uptake

**FIGURE 4:**
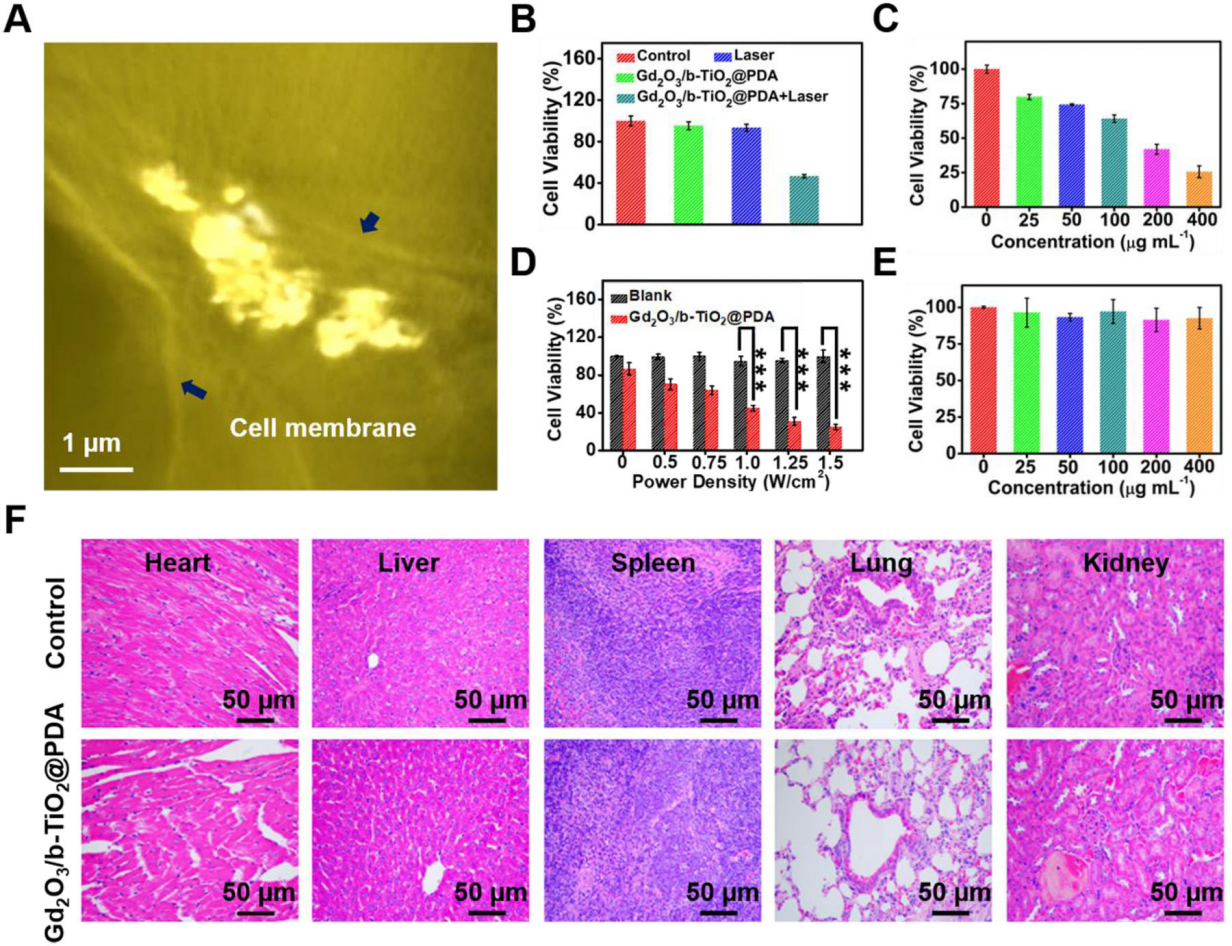
(A) Soft X‐ray 3D imaging of Gd_2_O_3_/b‐TiO_2_@PDA incubated with KYSE‐150 cells. (B) Cell viability of KYSE‐150 after different treatments. (C) Cell viability of KYSE‐150 incubated with different concentrations of Gd_2_O_3_/b‐TiO_2_@PDA under 1 W cm^−2^ 1064 nm laser irradiation for 5 min. (D) Cell viability of KYSE‐150 incubated with Gd_2_O_3_/b‐TiO_2_@PDA (b‐TiO_2_: 200 μg mL^−1^) under 1064 nm laser irradiation with various power densities for 5 min (mean ± standard, *n* = 5). Statistically significant differences were evaluated using the Student's *t*‐test (*** *p* < 0.001). (E) Viabilities of KYSE‐150 cells incubated with various concentrations of Gd_2_O_3_/b‐TiO_2_@PDA. (F) Histological analysis of major organs of healthy mice with the treatment of Gd_2_O_3_/b‐TiO_2_@PDA and saline on day 14

The uptake of nanoprobes by esophageal cancer cells (KYSE‐150) was directly observed using a three‐dimension (3D) soft X‐ray microscopy. Due to the large size of a single KYSE‐150 cell, Video S1 presents the 3D reconstruction soft X‐ray absorption‐contrast imaging of a partial of tumor cell. A slice of X‐ray absorption image is shown in Figure 4A; the tumor cell membrane and nucleus boundaries were clearly visible in the picture, and the nanoprobes were located in the middle of the boundaries, indicating that the cell can effectively uptake the nanoprobes. Therefore, the results demonstrated that the nanoprobes can enter the esophageal cancer cells and realize the function of PTT.

### 2.5 In vitro photothermal performances

For studying the PTT capability of Gd_2_O_3_/b‐TiO_2_@PDA nanoprobes in vitro, the cell survival rate was evaluated on KYSE‐150 cells under corresponding conditions. As shown in Figure 4B–D, a single factor change will not have a major impact on the cell viability, which was basically as same as the control, but when the nanoprobes and laser coexist, the cell survival rate was only 40%, and the cell survival rate was linearly inversely related to the material concentration and laser power density. In addition, the live and dead cell staining results intuitively showed that the majority cells dead when treated with the laser and Gd_2_O_3_/b‐TiO_2_@PDA (Figure S8). In summary, efficient tumor killing can be achieved under the combination of 1064 nm laser irradiation and nanoprobes, indicating that the nanoprobes had excellent PTT performance in vitro.

### 2.6 In vitro and in vivo biocompatibility

The cytotoxicity of Gd_2_O_3_/b‐TiO_2_@PDA nanoprobes was evaluated by MTT method. The results are shown in Figure 4E, when the concentration of the nanoprobes were 0, 25, 50, 100, 200, and 400 μg mL^−1^; the corresponding cell survival rates were 100%, 96.37%, 93.24%, 97.12%, 91.36%, and 92.51%, respectively. When the concentration of the nanoprobes was 400 μg mL^−1^, the cell survival rate was about 90%, indicating that the nanoprobes caused no obvious damage to the cells and had good biocompatibility in vitro.^[^ ^28^ ^]^

The biocompatibility of the Gd_2_O_3_/b‐TiO_2_@PDA nanoprobes in vivo was investigated through the histological analysis. The tissue sections of the main organs (heart, liver, spleen, lung, and kidney) were collected after treatment for the H &E staining. As shown in Figure 4F, the staining results showed the Gd_2_O_3_/b‐TiO_2_@PDA had no obvious damage to the tissue, indicating the nanoprobes had appreciable biocompatibility in vivo. To further analyze the safety of the nanoprobes, the blood was collected after treatment for hematological analysis and blood biochemical analysis. As the results shown in Figure S9, the mice treated with Gd_2_O_3_/b‐TiO_2_@PDA and saline had no significant change in the indicators of hematological and blood biochemical analysis, revealing that the nanoprobes having good biocompatibility ensure its possibility of clinical application.

### 2.7 In vivo MR imaging and photothermal therapy

**FIGURE 5:**
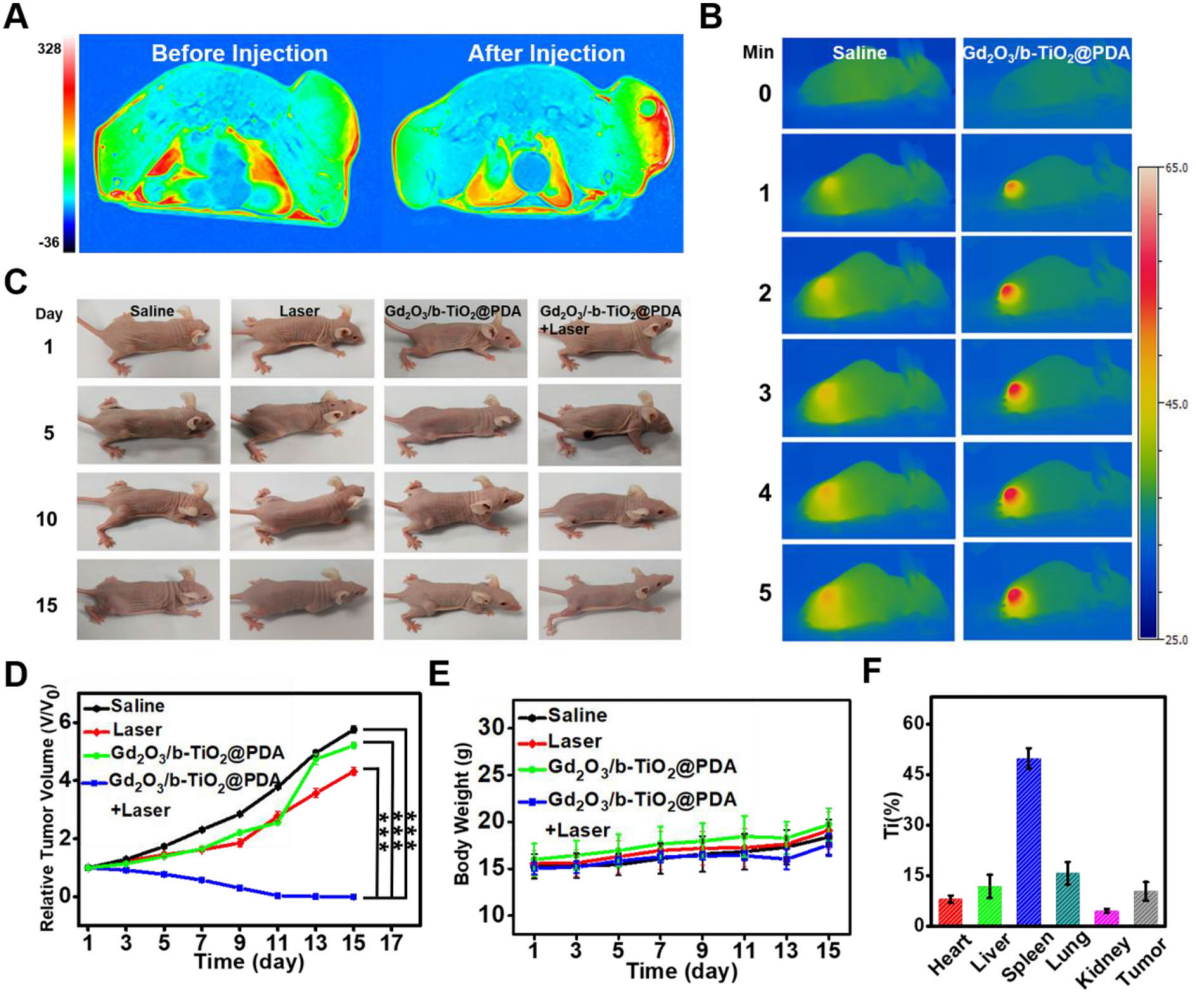
(A) T_1_‐weighted MR images on the KYSE‐150 tumor‐bearing mice before and after in situ injection of Gd_2_O_3_/b‐TiO_2_@PDA. (B) Photothermal images of KYSE‐150 tumor‐bearing mice under irradiation at varied time‐points. (C) Photographs of KYSE‐150 tumor‐bearing mice at different days during the treatment. The relative tumor volumes (D) and the body weights (E) in different treating groups of tumor‐bearing mice. (F) Biodistribution of Ti in main organs and tumor from KYSE‐150 tumor‐bearing mice at 24 h after intravenous injection of Gd_2_O_3_/b‐TiO_2_@PDA (mean ± standard, *n* = 5). Statistically significant differences were evaluated using the Student's *t*‐*t*est (*** *p* < 0.001)

In order to measure the MR imaging performance of the nanoprobes in vivo, T_1_‐weighted MR imaging was performed on the KYSE‐150 tumor‐bearing nude mice by using a 3.0 T MAGNETOM Prisma MR device. Figure 5A shows that there was no visibly signal before injection, but a strong red signal appeared after injection, and the red signal corresponded to the tumor area. A small hole appeared in Figure 5A, caused by the rapid diffusion of the probe inside the tumor. Experimental results showed that the Gd_2_O_3_/b‐TiO_2_@PDA nanoprobes possessed excellent T_1_ enhanced MR imaging performance in vivo. The thermal imaging of mice treated with the nanoprobes and saline was significantly different, indicating the nanoprobes exhibited considerable photothermal performance and combined with MR imaging can provide a dual‐modal imaging system (Figure 5B).

To evaluate the photothermal therapeutic efficiency in vivo, relevant parameters of mice were collected every 2 days. As shown in Figure 5C, the relative volume of tumor in the PTT group gradually decreased in the treatment period, and disappeared on the 11^th^ day. However, the relative volume of tumor in other groups gradually increased (Figure 5D), which was consistent with the pictures of the tumor‐bearing mouse shown in Figure 5C. The experimental results indicated that the nanoprobes had significant effect on photothermal treatment in vivo. Meanwhile, the results in Figure 5E show that the body weight change curve of the mice in each group presented an upward trend with time prolonged, indicating the nanoprobes were considerably safe and will not affect the health of mice. Furthermore, the results of the metabolic distribution of the nanoprobes in vivo showed that the nanoprobes were most distributed in the spleen and least in the kidney, minimizing the risk of nephrogenic side effects, which further pointed to the safety of probes (Figure 5F). The excellent MR imaging and PTT treatment performance of the probes realized the safety and efficient therapy of esophageal cancer, and provided certain theoretical guidance for clinical research.

## 3 CONCLUSION

A novel NIR region laser‐induced composite nanoprobes Gd_2_O_3_/b‐TiO_2_@PDA was successfully constructed for MR imaging‐guided NIR‐II PTT in this study. This PDA‐coated b‐TiO_2_ nanoprobe had not only solved the problems of shallow tissue penetration depth with 1064 nm laser irradiation, but also had high photothermal conversion efficiency as high as 64.9% under an irradiation of 1 W cm^−2^, which does not exceed the maximum permissible exposure of skin. In addition, the photothermal mechanism of the Gd_2_O_3_/b‐TiO_2_@PDA composite nanoprobes was fully explained. First, the band gap of b‐TiO_2_ was greatly reduced to 1.1 eV by introducing more oxygen vacancies and Ti^3+^ that the Gd_2_O_3_/b‐TiO_2_@PDA nanoprobes showed enhanced 1064 nm light absorption. More importantly, compared with pure b‐TiO_2_, the synergistic effect of the PDA π molecular orbital highly electronically delocalized and the b‐TiO_2_ similar to the metal localized surface plasmon resonance facilitated the transfer of charge carriers, thus leading to the dramatic improvement of NIR‐II light‐driven photothermal conversion performance. Meanwhile, the component of Gd_2_O_3_ endowed the nanoprobes with excellent T_1_‐weighted MR imaging performance that implements MR/thermal dual‐modal image‐guided PTT toward esophageal cancer cell. For further application, in the model of esophageal cancer, the tumor completely disappeared when treated with Gd_2_O_3_/b‐TiO_2_@PDA at the 11^th^ day, demonstrating the nanoprobes can serve as a safe and efficient diagnostic and therapeutic agent, and provided certain theoretical guidance and experience for clinical research.

## 4 EXPERIMENTAL SECTION

Experimental details are provided in the Supporting Information.

## CONFLICT OF INTEREST

Aiguo Wu is a member of the *Exploration* editorial board. The authors declare no conflict of interest.

## ETHICS STATEMENT

All the animal procedures were performed in compliance with the Regulations for the Administration of Affairs Concerning Experimental Animals of China, and all animal experiments were approved by the Regional Ethics Committee for Animal Experiments of the Ningbo University (Permit No. SYXK (Zhe) 2019‐0005).

## Supporting information



Supporting Information

Supplemental Video 1

## Data Availability

All data associated with this study are present in the paper or in the Supporting Information.
