# Supplementary material for: Gd2O3/b‐TiO2 composite nanoprobes with ultra‐high photoconversion efficiency for MR image‐guided NIR‐II photothermal therapy
Source: Exploration (Beijing). 2022 Jun 13;2(6):20220014. doi: 10.1002/EXP.20220014 (PMC10190978; doi:10.1002/EXP.20220014)
Supplement: Supplementary file 1 — Supporting Information [file EXP2-2-20220014-s002.docx]

Supporting information

### Gd_2_O_3_/b-TiO_2_ composite nanoprobes with ultra-high photoconversion efficiency for MR image-guided NIR-II photothermal therapy

Jia Chen^1,2^ | Tianxiang Chen^1,4^ | Qianlan Fang^1,5^ | Chunshu Pan^3^ | Ozioma Udochukwu Akakuru^1,5^ | Wenzhi Ren^1,4^ | Jie Lin^1,4^ | Aizhu Sheng^3^ | Xuehua Ma^1,4,5^ | Aiguo Wu^1,4^

^1^ Cixi Institute of Biomedical Engineering, International Cooperation Base of Biomedical Materials Technology and Application, Chinese Academy of Science (CAS) Key Laboratory of Magnetic Materials and Devices and Zhejiang Engineering Research Center for Biomedical Materials, Ningbo Institute of Materials Technology and Engineering, CAS, Ningbo, P.R. China.

^2^ School of Life Science and Engineering, Southwest Jiaotong University, Chengdu, P.R. China.

^3^ Department of Radiology, Hwa Mei Hospital, University of Chinese Academy of Sciences, Ningbo, P.R. China.

^4^ Advanced Energy Science and Technology Guangdong Laboratory, Huizhou, P.R. China.

^5^ University of Chinese Academy of Sciences, No. 1 Yanqihu East Road, Huairou District, Beijing, P.R. China.

**Correspondence**

Aiguo Wu, Cixi Institute of Biomedical Engineering, International Cooperation Base of Biomedical Materials Technology and Application, Chinese Academy of Science (CAS) Key Laboratory of Magnetic Materials and Devices and Zhejiang Engineering Research Center for Biomedical Materials, Ningbo Institute of Materials Technology and Engineering, CAS, Ningbo 315201, P.R. China.

Email: aiguo@nimte.ac.cn

Xuehua Ma, Cixi Institute of Biomedical Engineering, International Cooperation Base of Biomedical Materials Technology and Application, Chinese Academy of Science (CAS) Key Laboratory of Magnetic Materials and Devices and Zhejiang Engineering Research Center for Biomedical Materials, Ningbo Institute of Materials Technology and Engineering, CAS, Ningbo 315201, P.R. China.

Email: maxh@nimte.ac.cn

Aizhu Sheng, Department of Radiology, Hwa Mei Hospital, University of Chinese Academy of Sciences, Ningbo 315010, P.R. China.

Email: 283647666@qq.com

Jia Chen and Tianxiang Chen contributed equally to this work.

**EXPERIMENTAL SECTION**

**Materials**

P25 was purchased from Acros Organics (Beijing, China). Sodium borohydride (NaBH_4_), gadolinium nitrate hexahydrate (Gd (NO_3_)_3_·6H_2_O), sodium hydroxide (NaOH), diethylene glycol (DEG), 3-Hydroxytyramine hydrochloride (DA·HCl), dimethyl sulfoxide (DMSO), 3-(4,5-dimethylthiazol-2-yl)-2,5-diphenyltetrazolium bromide (MTT), and chloral hydrate (CCI_3_CH(OH)_2_) were purchased from Aladdin (Shanghai, China). Absolute ethanol, ammonia solution (NH_3_·H_2_O), and Agar were purchased from Sinopharm (Shanghai, China). Fetal bovine serum (FBS), trypsin, and Roswell Park Memorial Institute (RPMI) were purchased from Keygen (Nanjing, China). All the agents were used without further purification. Deionized water (Millipore Milli-Q grade, 18.2 MΩ) was used in the following experiments.

**Synthesis of Gd_2_O_3_/b-TiO_2_@PDA**

b-TiO_2_ nanoparticles were prepared through a controllable solid-state reaction of NaBH_4_ and crystalline P25 according to a previous report.^[1]^ Simply, 1.5 g of P25 was mixed with 1.5 g of NaBH_4_ and ground adequately for 30 minutes in a mortar. Then the mixture was transferred into a porcelain boat, which was put into a tubular furnace and reacted at 350 °C for 3 h under Ar atmosphere. After the reaction, the product was placed in deionized water overnight to remove excess NaBH_4_. Finally, the b-TiO_2_ nanoparticles were obtained by centrifugation, washing [alternately](javascript:;) with deionized water/ethanol (v/v = 1/1) for four times, and drying at 70 °C in a vacuum drying oven.

Gd_2_O_3_/b-TiO_2_ nanoparticles were prepared by one-pot reaction on the basis of a polyol method.^[2]^ Specifically, 0.906 g of NaOH was dissolved in 90.6 mL diethylene glycol (DEG), and 0.36 g of Gd (NO_3_)_3_·6H_2_O was added to the solution. Meanwhile, 160 mg of b-TiO_2_ was dissolved in 40 mL DEG and dispersed uniformly through a cell breaker. After that, the solutions were mixed and diluted with DEG to 160 mL, transferred to 250 mL three-neck flask, and refluxed at 140 ℃ for 1 h. Thereafter, the reaction condition was raised to 175 ℃ for another 4 h. Finally, the Gd_2_O_3_/b-TiO_2_ nanoparticles were collected and washed with deionized water/ethanol (v/v = 1/1) for eight times by centrifugation and dispersed in 20 mL deionized water and stored at 4 ℃ for the following experiments.

Gd_2_O_3_/b-TiO_2_@PDA nanoparticles were obtained by oxidative self-aggregation of DA·HCl.^[3]^ Specifically, 4.5 mL Gd_2_O_3_/b-TiO_2_ solution (30 mg b-TiO_2_) was dispersed in 85 mL deionized water and 40 mL absolute ethanol, followed by the addition of 3 mL NH_3_·H_2_O stirring at room temperature for 30 min. Thereafter, 10 mL DA·HCl (15 mg mL^-1^) was added to the above solution and stirred for 8 h. The Gd_2_O_3_/b-TiO_2_@PDA nanoparticles were obtained by centrifugation, washed with deionized water/ethanol (v/v = 1/1) for four times to remove excess unreacted DA·HCl, and dispersed in 10 mL deionized water for further use.

**Characterization of nanoparticles**

The morphologies of nanoparticles were determined using JEOL2100 transmission electron microscope (TEM) (Japan Electronics Co. Ltd, Japan). High-resolution TEM (HRTEM), high-angle annular dark-field scanning TEM (HAADF-STEM), and energy-dispersive X-ray spectroscopy (EDS) line scanning images were collected on Tecnai F20 (FEI, USA). X-ray powder diffractometer (XRD) patterns were acquired on a D8 ADVANCE (BRUKER, [Germany](javascript:;)). X-ray photoelectron spectroscopy (XPS) was performed on an Axis Ultra DLD instrument (Kratos, UK). The particle size and zeta potential measurements were conducted by dynamic light scattering (DLS) using a Zetasizer Nano ZS system (Malvern, UK).

Nicolet 6700 Fourier transform infrared (FT-IR) spectrometer (Thermo scientific, USA) and Lambda 950 UV-vis spectrophotometer (PerkinElmer, USA) was used to obtain the functional groups and determine light absorption of the nanoparticles. A SPECTRO ARCOSⅡ analytical instrument (SPECTRO, Germany) was used to perform the element content analysis by inductively coupled plasma optical emission spectrometry (ICP-OES). A MesoMR23-060H-1 MR scanner system (NIUMAG, China) was used to acquire the MR imaging and relaxation performance of the nanoparticles. A 1064 nm multimode pump laser (Xian Electronic Technology Co. Ltd.) was used as the irradiation source for photothermal hyperthermia.

**Photothermal performance of nanoparticles**

To evaluate the photothermal performance in vitro, changes in temperature were monitored by an photothermal imager upon irradiation with a 1064 nm laser. The specific experiments are shown as follows: (1) Photothermal properties of different materials: b-TiO_2_, Gd_2_O_3_/b-TiO_2_, and Gd_2_O_3_/b-TiO_2_@PDA with a series of concentrations (0, 50, 100, 200, and 400 μg mL^-1^ based on b-TiO_2_) were irradiated by 1064 nm laser for 5 min at a power density of 1 W cm^-2^. (2) Photothermal properties at different power densities: Gd_2_O_3_/b-TiO_2_@PDA (200 μg mL^-1^ based on the b-TiO_2_) with a series of power densities (0.5, 0.75, 1, 1.25, and 1.5 W cm^-2^) was irradiated by 1064 nm laser for 5 min. (3) Photothermal stability: Gd_2_O_3_/b-TiO_2_@PDA (200 μg mL^-1^ based on the b-TiO_2_) was irradiated by 1064 nm laser (1 W cm^-2^) for five on/off cycles at 5 min laser ‘on’ and ‘off’ in each case. (4) Photothermal conversion efficiency: Gd_2_O_3_/b-TiO_2_@PDA (200 μg mL^-1^ based on the b-TiO_2_) was irradiated by 1064 nm laser (1 W cm^-2^) for 10 min and naturally cooled to room temperature. The photothermal conversion efficiency can be calculated from the ascending and descending curve.

**In vitro MR imaging and relaxation properties**

In order to acquire the MR imaging performance, a 0.5 T MesoMR23-060H-1 MR scanner was used. Specifically, different concentrations of Gd_2_O_3_/b-TiO_2_@PDA were set based on the gadolinium element (0, 0.125, 0.25, 0.5, 1, and 2 mM). These solutions were transferred to 1 mL centrifuge tubes and scanned with the MR machine. The specific parameters were as follows: T_1_ spin echo sequence, TR = 300 ms, TE = 18.2 ms, slice width = 3 mm, FOV = 100 mm × 100 mm. Meanwhile, the longitudinal relaxation times (T_1_) and transverse relaxation times (T_2_) were measured, and the longitudinal relaxivity (r_1_) and transverse relaxivity (r_2_) were obtained from the slope of the relaxation rate r_1_ (1/T_1_) and r_2_ (1/T_2_), respectively.

**Cell culture**

Esophageal squamous cell carcinoma cells (KYSE-150) were purchased from Zhong Qiao Xin Zhou Biotechnology Co. Ltd (Shanghai, China), and cultured with 1640 complete medium, which contained 10% fetal bovine serum (FBS). In addition, the KYSE-150 cells were cultured in the humidified incubator with a partial pressure of 5 % CO_2_ at 37 °C and passaged every 2 days.

**Cellular uptake**

KYSE-150 cells were seeded in 6-well plates for 12 h and the density was 3 × 10^5^ cells per well. Then, the culture medium was replaced with 100 μL of Gd_2_O_3_/b-TiO_2_@PDA (200 μg mL^-1^ based on the b-TiO_2_) for another 12 h. After that, the mixture was removed, and cells were washed with PBS for 3 times and stained with 4 % polyformaldehyde for 30 min. In the end, the cells were washed with PBS and stored in PBS for next use. The prepared samples were measured by the 3D soft X-ray imaging of Hefei light source (Hefei, China), which can provide the results of cellular uptake through three-dimensional image reconstruction.

**In vitro photothermal therapy**

The standard MTT assay was used to measure photothermal therapy in vitro. Specifically, KYSE-150 cells were seeded in 96-well plates for 12 h and the density was 1 × 10^4^ cells per well. Then, the culture medium was replaced with 100 μL of Gd_2_O_3_/b-TiO_2_@PDA (200 μg mL^-1^ based on the b-TiO_2_) for another 4 h. The wells were divided into four groups, including control group, laser only group, Gd_2_O_3_/b-TiO_2_@PDA only group, and Gd_2_O_3_/b-TiO_2_@PDA and laser group. In addition, KYSE-150 cells were incubated with Gd_2_O_3_/b-TiO_2_@PDA and irradiated by 1064 nm laser for 5 min with different power densities (0, 0.5, 0.75, 1, 1.25, and 1.5 W cm^-2^). Meanwhile, KYSE-150 cells were incubated with Gd_2_O_3_/b-TiO_2_@PDA of different concentrations (0, 25, 50, 100, 200, and 400 μg mL^-1^) and irradiated by 1064 nm laser for 5 min at 1 W cm^-2^ power density.

**In vitro and in vivo biocompatibility**

In order to evaluate the cytotoxicity of Gd_2_O_3_/b-TiO_2_@PDA nanoparticles in vitro, a standard MTT viability assay was adopted. Briefly, KYSE-150 cells were seeded in 96-well plates for 12 h with a density of 1 × 10^4^ cells per well and then cultured with 100 μL of Gd_2_O_3_/b-TiO_2_@PDA (0, 25, 50, 100, 200, and 400 μg mL^-1^ based on the b-TiO_2_) for 20 h. After that, 10 μL of MTT (5 mg mL^-1^) was added to the above wells and incubated for 4 h. Finally, the MTT solutions were removed and 100 μL of DMSO was added to 96-well plates. The absorbance was measured by a microplate reader (imark 168-1130, USA) and the corresponding wavelength is 490 nm. The cell viability of KYSE-150 can be calculated from the above results.

For the biocompatibility test in vivo, 100 μL of saline and Gd_2_O_3_/b-TiO_2_@PDA (4 mg mL^-1^) were injected into healthy nude mice via the tail vein. After feeding for 7 days, the mice blood was stored in anticoagulation and coagulation tubes by taking blood from the eyeball. Subsequently, the blood in the coagulation tube was further centrifuged (3000 rpm, 10 min), the upper serum was taken out and packaged, and finally all the blood samples obtained were sent to the blood testing center for blood routine and blood biochemical analysis. The safety of the nanoprobes was judged by the injection of physiological saline and the blood indicators of the mice injected with the nanoprobes. After 14 days of normal rearing, the mice were sacrificed, and their main organs (heart, liver, spleen, lung, and kidney) were harvested for hematoxylin/eosin (H&E) staining, and then the tissue structure was observed and photographed using an optical microscope.

**In vivo MR imaging and photothermal therapy**

Balb/C female nude mice (3-6 weeks) were purchased from Suzhou Cavens Biogle Model Animal Research Co., Ltd (China). All the animal experiments were performed in compliance with the Regulations for the Administration of Affairs Concerning Experimental Animals of China, and the protocols were approved by the Regional Ethics Committee for Animal Experiments at Ningbo University, China.

For the MR imaging in vivo, 100 μL (calculated by Gd: 0.5 mM) Gd_2_O_3_/-TiO_2_@PDA solution was injected in situ into the KYSE-150 tumor-bearing nude mice. A 3.0 T MAGNETOM Prisma MR equipment was used to collect T_1_-weighted MR imaging grayscale images before and 30 min after injection, and the data were imported into eWorldView image processing software for subsequent optimization. The images were compared before and after injection to assess the in vivo imaging effect.

As regards photothermal therapy in vivo, the successfully modeled nude mice were randomly divided into four groups (n=5): saline, laser, Gd_2_O_3_/b-TiO_2_@PDA, Gd_2_O_3_/b-TiO_2_@PDA + laser, in which 100µL of saline, saline, Gd_2_O_3_/b-TiO_2_@PDA (200 μg mL^-1^ based on the b-TiO_2_) and Gd_2_O_3_/b-TiO_2_@PDA (200 μg mL^-1^ based on the b-TiO_2_) was injected in situ, respectively. Then the mice were immediately anesthetized with 8 wt% chloral hydrate solution by intraperitoneally injection and the tumor irradiated with or without 1064 nm laser at 1 W cm^-2^ for 5 min. After treatment, the tumor size was measured by digital caliper. Tumor volume (V) = length × width^2^/2. Relative tumor volume was calculated as V/V0 (V0 was the corresponding tumor volume when the treatment was initiated). The body weights were also recorded for every two days. The collected data were classified and summarized, and their respective change curves were drawn to evaluate the treatment effect from the changes in the curves.

**REFERENCE**

[1] Y. Gao, L. Zhang, Y. Liu, S. Sun, Z. Yin, L. Zhang, A. Li, G. Lu, A. Wu, L. Zeng, *Nanoscale* **2020**, *12*, 1801.

[2] X. Ma, A. Gong, L. Xiang, T. Chen, Y. Gao, X. Liang, Z. Shen, A. Wu, *J. Mater. Chem. B*. **2013**, *1*, 3419.

[3] H. S. Jung, K. J. Cho, Y. Seol, Y. Takagi, A. Dittmore, P. A. Roche, K. C. Neuman, *Adv. Funct. Mater*. **2018**, *28*, 1801252.

**
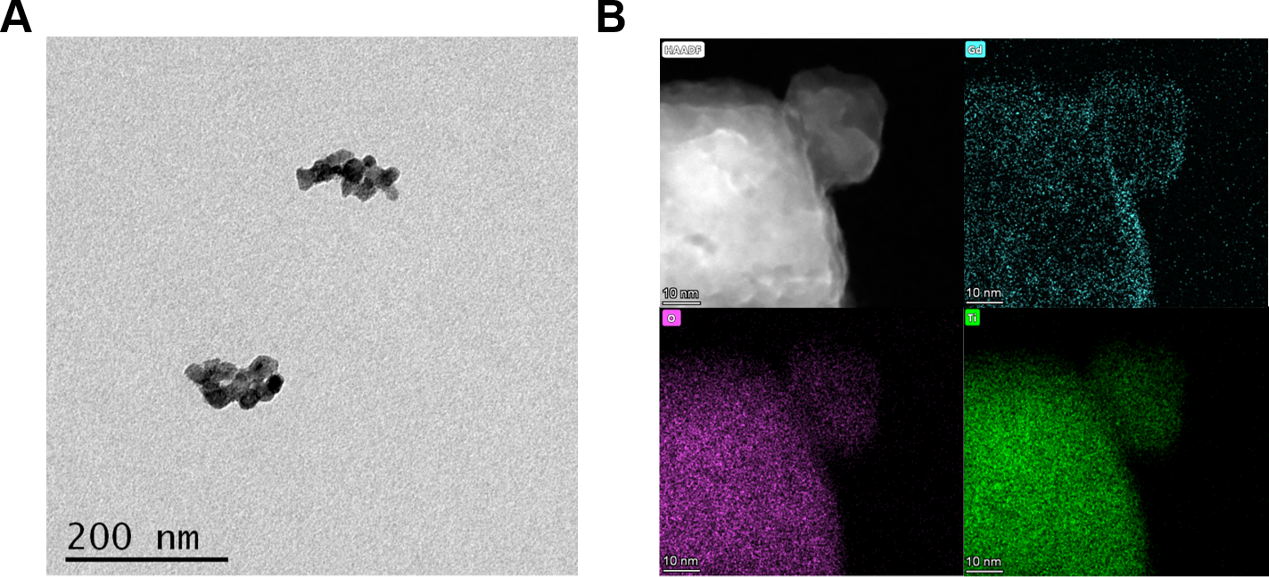
**

**Figure S1** (A)TEM image of Gd_2_O_3_/b-TiO_2_. (B) STEM-HAADF images of Gd_2_O_3_/b-TiO_2_ and the corresponding EDS elemental mappings.


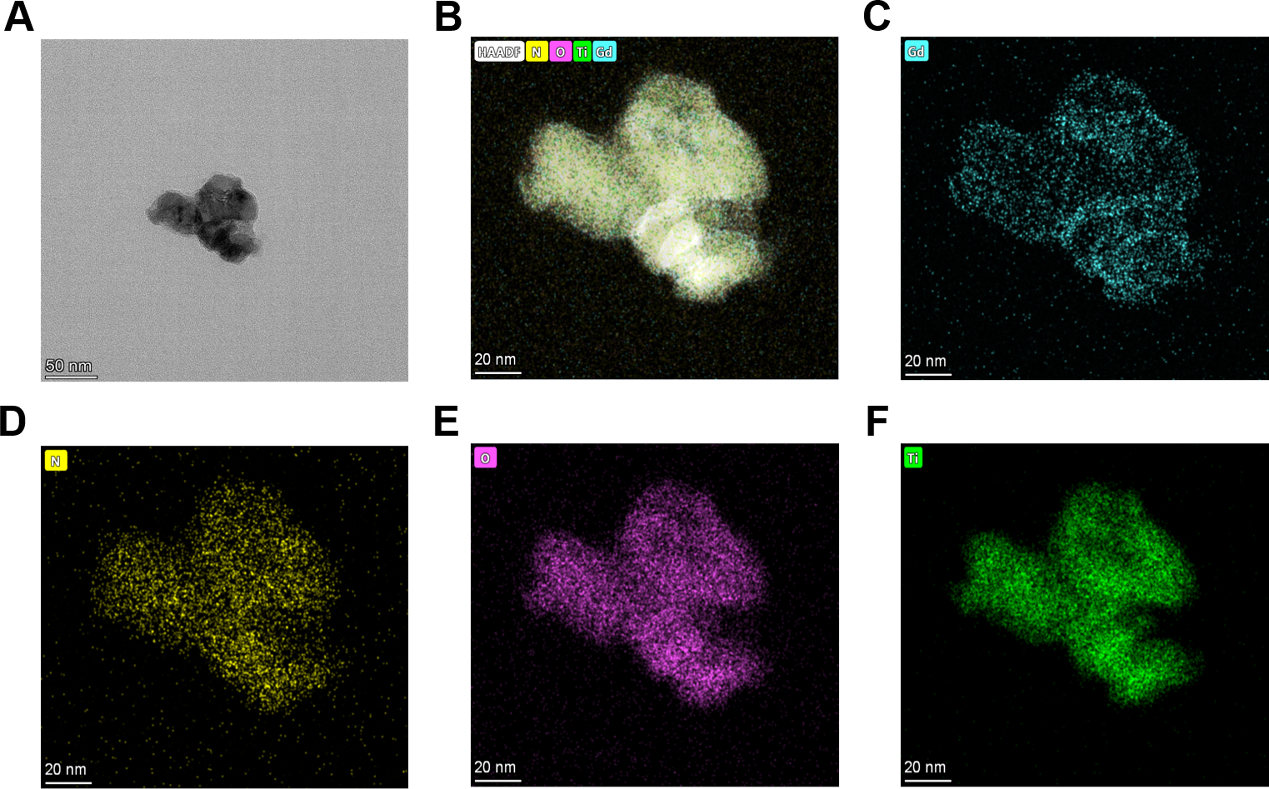


**Figure S2** STEM-HAADF images of Gd_2_O_3_/b-TiO_2_@PDA and the corresponding EDS elemental mappings.

**
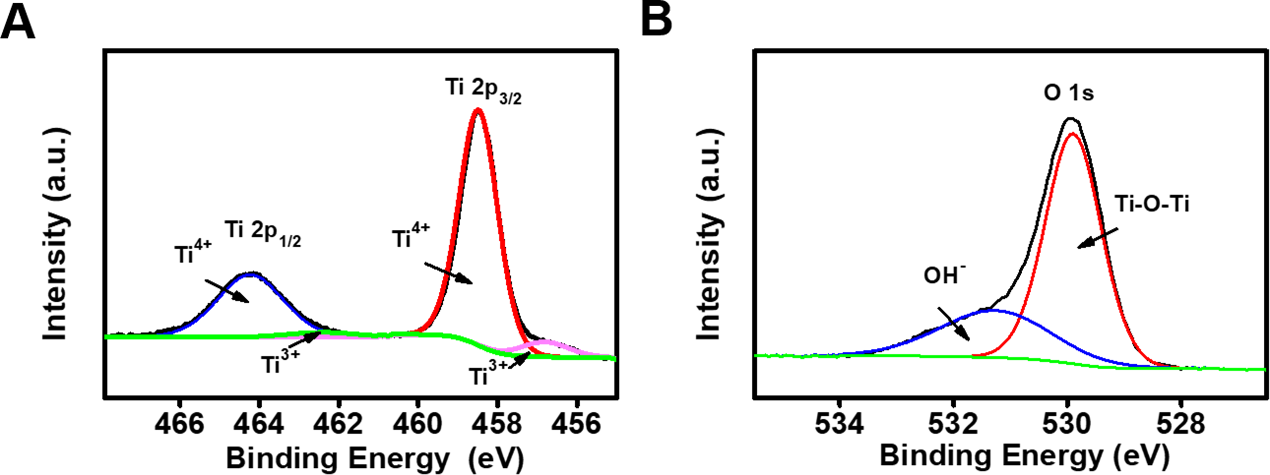
**

**Figure S3** XPS patterns of (A) Ti 2p and (B) O 1s in b-TiO_2_.


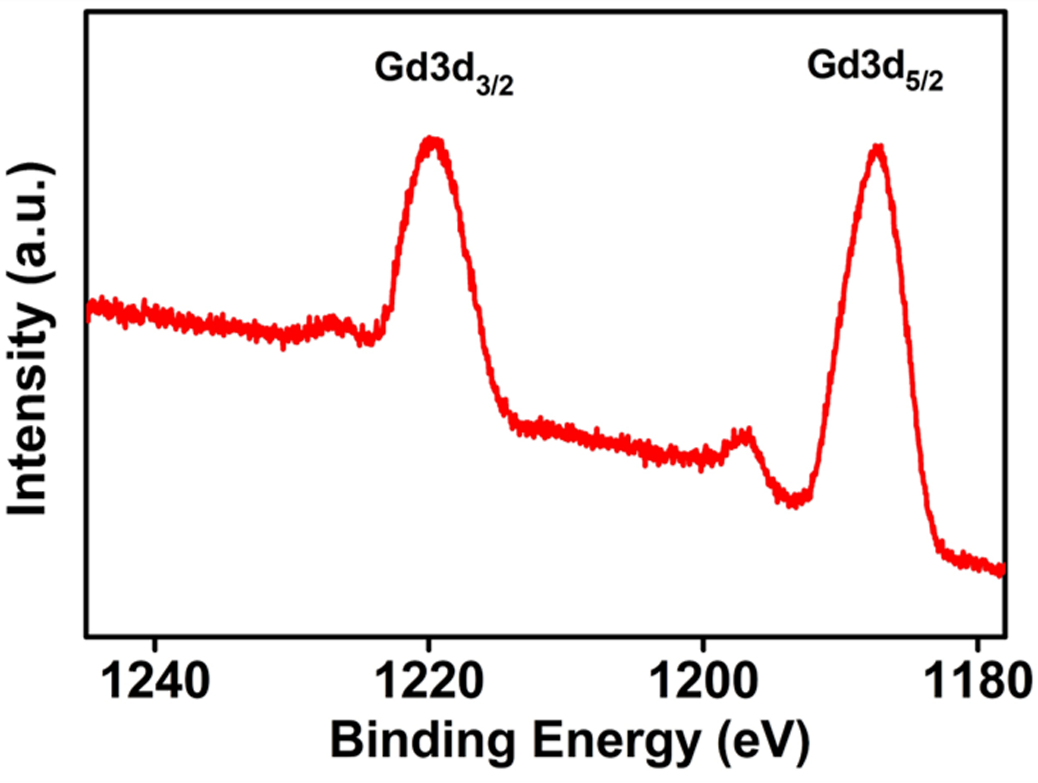


**Figure S4** XPS pattern of Gd 3d in Gd_2_O_3_/b-TiO_2_.


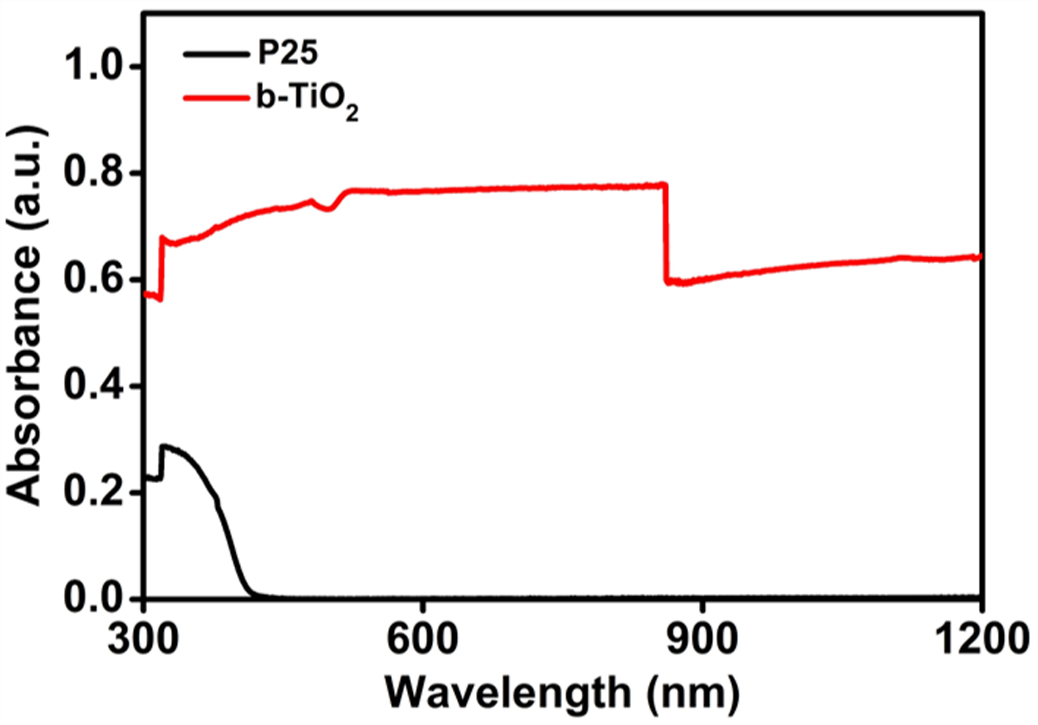


**Figure S5** The diffuse reflectance UV-Vis spectra of P25 and b-TiO_2_.

**
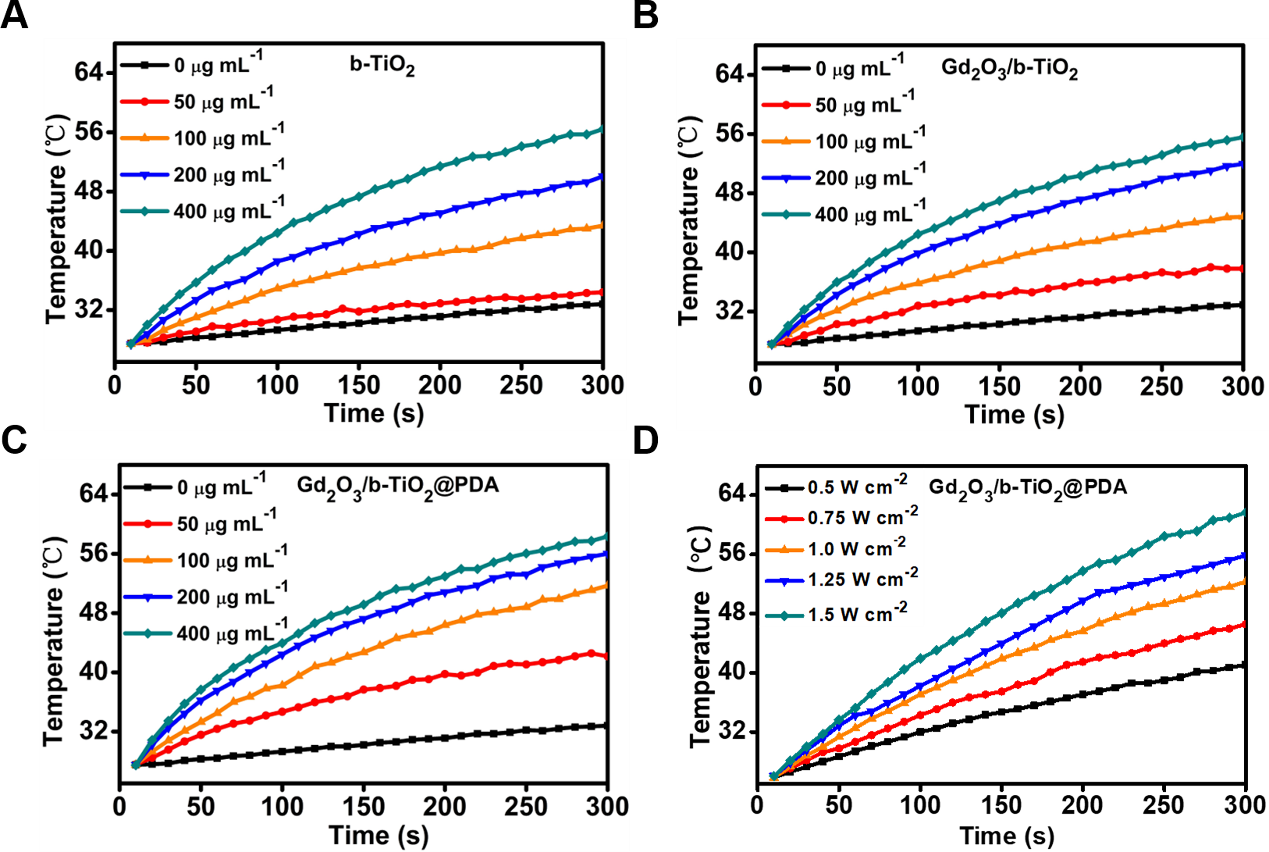
**

**Figure S6** Temperature change curves of (A) b-TiO_2_, (B) Gd_2_O_3_/b-TiO_2_, and (C) Gd_2_O_3_/b-TiO_2_@PDA with various concentrations under 1 W cm^-2^ 1064 nm laser irradiation for 5 min. (D) Temperature change curves of Gd_2_O_3_/b-TiO_2_@PDA (b-TiO_2_: 200 μg mL^-1^) under 1064 nm laser irradiation with various power density.


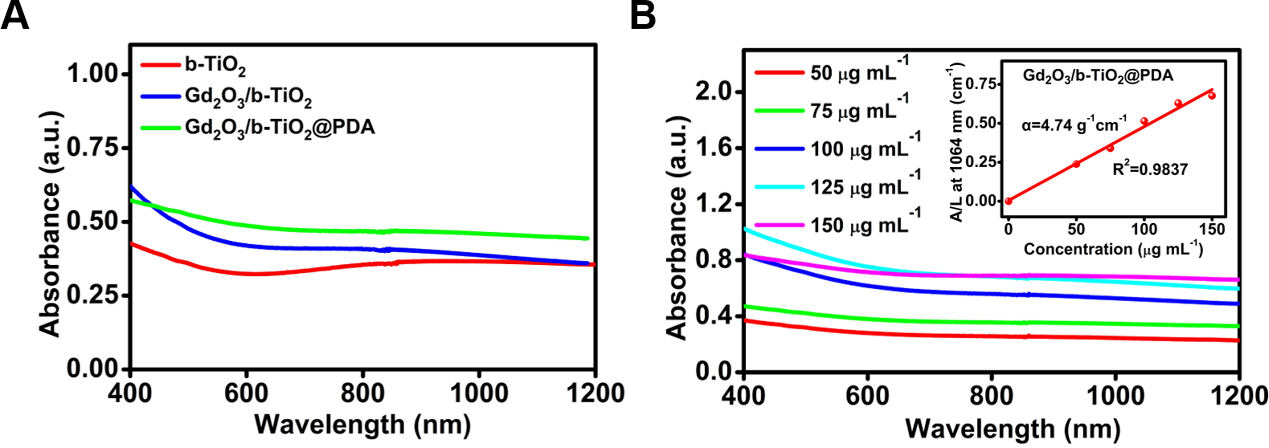


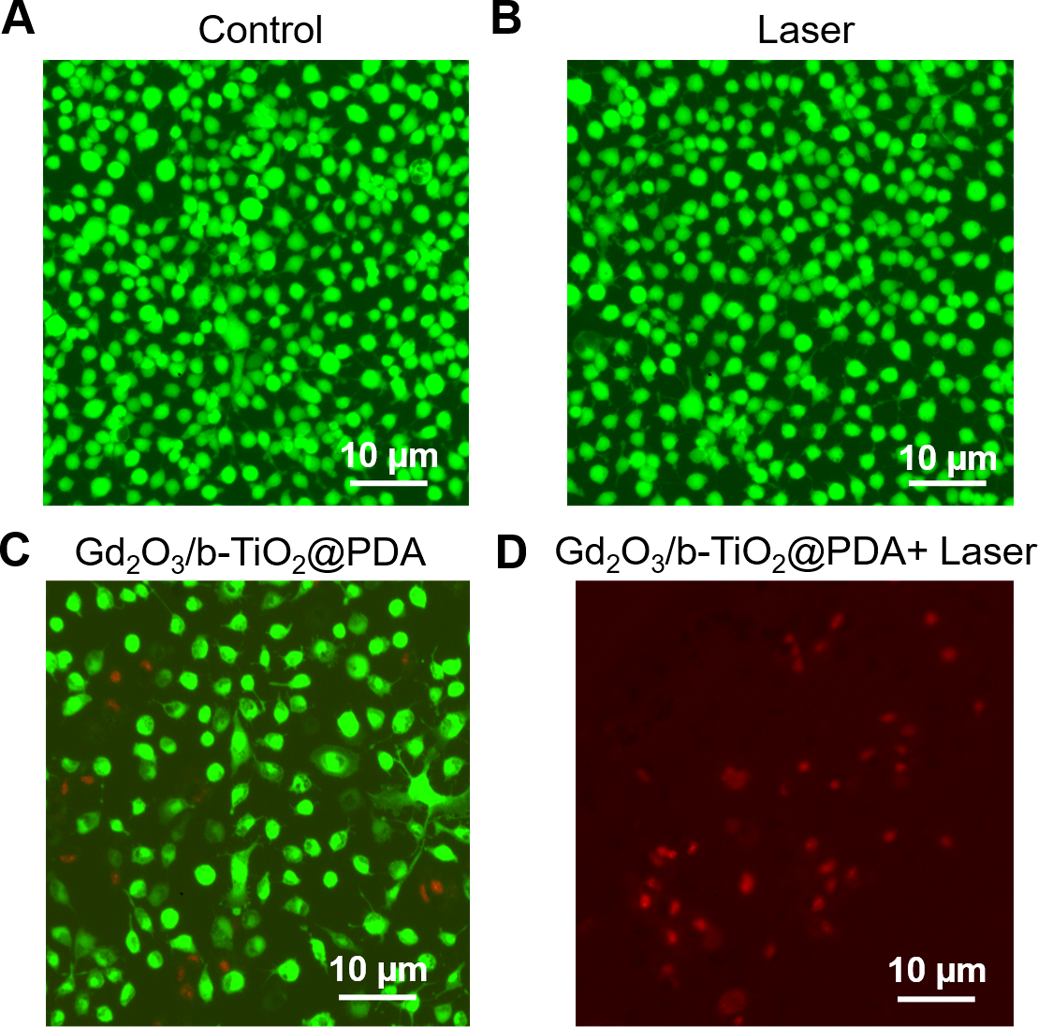
**Figure S7** (A) UV-vis spectra of b-TiO_2_, Gd_2_O_3_/b-TiO_2_ and Gd_2_O_3_/b-TiO_2_@PDA. (B) UV-vis spectra of Gd_2_O_3_/b-TiO_2_@PDA with different concentrations. Inset: Mass extinction coefficient of Gd_2_O_3_/b-TiO_2_@PDA at 1064 nm.

**Figure S8** Microscope images of calcein AM (green, live cells) and propidium Iodide (red, dead cells) co-stained KYSE-150 cells treated with or without Gd_2_O_3_/b-TiO_2_@PDA and laser irradiation for 5 min.

**
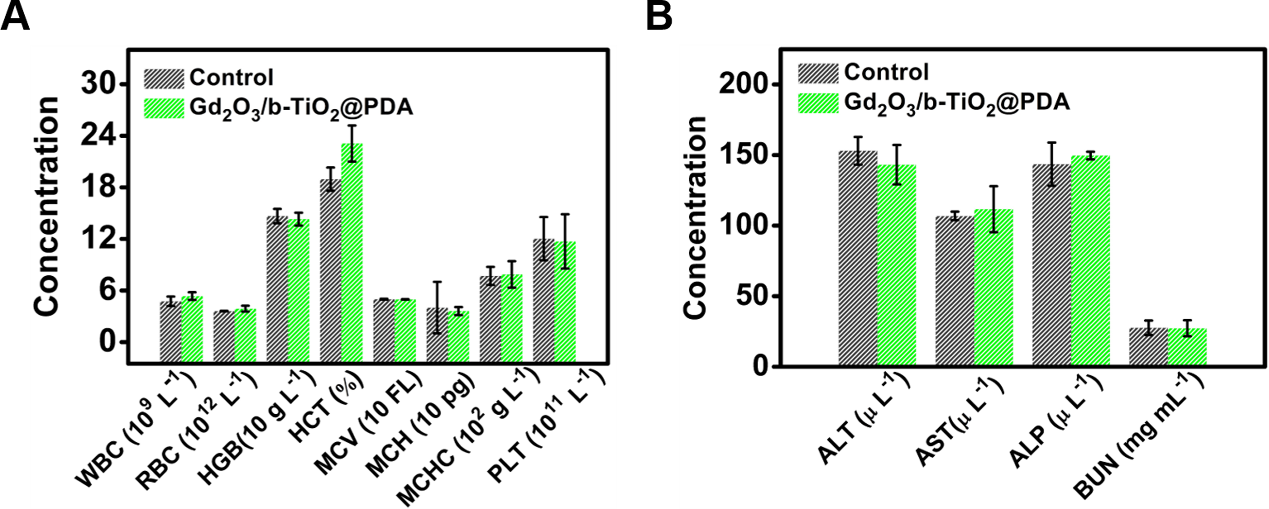
Figure S9** (A) Hematological analysis and (B) blood biochemical analysis of the mice, which were injected with saline and Gd_2_O_3_/b-TiO_2_@PDA through the tail vein. Data are expressed as the mean ± standard (n = 3).
